# Radiomics Study for Differentiating Focal Hepatic Lesions Based on Unenhanced CT Images

**DOI:** 10.3389/fonc.2022.650797

**Published:** 2022-04-27

**Authors:** Xitong Zhao, Pan Liang, Liuliang Yong, Yan Jia, Jianbo Gao

**Affiliations:** ^1^Department of Radiology, The First Affiliated Hospital of Zhengzhou University, Zhengzhou, China; ^2^Scientific Research Department, Huiying Medical Technology Co., Ltd, Beijing, China

**Keywords:** liver neoplasms, multidetector computed tomography, diagnosis, computer-aided design, radiomics, medical oncology

## Abstract

**Objectives:**

To investigate the feasibility of computer-aided discriminative diagnosis among hepatocellular carcinoma (HCC), hepatic metastasis, hepatic hemangioma, hepatic cysts, hepatic adenoma, and hepatic focal nodular hyperplasia, based on radiomics analysis of unenhanced CT images.

**Methods:**

452 patients with 77 with HCC, 104 with hepatic metastases, 126 with hepatic hemangioma, 99 with hepatic cysts, 24 with FNH, 22 with HA, who underwent CT examination from 2016 to 2018, were included. Radcloud Platform was used to extract radiomics features from manual delineation on unenhanced CT images. Most relevant radiomic features were selected from 1409 *via* LASSO (least absolute shrinkage and selection operator). The whole dataset was divided into training and testing set with the ratio of 8:2 using computer-generated random numbers. Support Vector Machine (SVM) was used to establish the classifier.

**Results:**

The computer-aided diagnosis model was established based on radiomic features of unenhanced CT images. 27 optimal discriminative features were selected to distinguish the six different histopathological types of all lesions. The classifiers had good diagnostic performance, with the area under curve (AUC) values greater than 0.900 in training and validation groups. The overall accuracy of the training and testing set about differentiating the six different histopathological types of all lesions was 0.88 and 0.76 respectively. 34 optimal discriminative were selected to distinguish the benign and malignant tumors. The overall accuracy in the training and testing set was 0.89and 0.84 respectively.

**Conclusions:**

The computer-aided discriminative diagnosis model based on unenhanced CT images has good clinical potential in distinguishing focal hepatic lesions with noninvasive radiomic features.

## Introduction

In recent years, the incidence of focal hepatic lesions has gradually increased. Among them, the hepatic cyst is the most common benign liver tumor followed by hepatic hemangioma. Although ninety percent of malignant primary liver tumors are primary hepatocellular carcinoma (HCC), hepatic metastasis from various primaries occur 20 times more common than HCC and are often multifocal (1). Hepatic imaging is essential for preoperative diagnosis of patients with focal hepatic lesions, which is critical to the choice of proper treatment and patient prognosis (1–3). However, preoperative imaging diagnosis of benign and malignant focal hepatic lesions is difficult, and sometimes impossible, especially for atypical or multiple lesions (4, 5). Computed Tomography (CT) is the most commonly used method for diagnosing focal hepatic lesions (6). However, its diagnostic accuracy cannot be compared with pathological examinations, especially for lesions with similar imaging features (7). Conventional CT examinations still have certain limitations, such as allergic reactions to contrast agents and certain radiation damage.

Radiomics is a newly emerging image analysis technology in recent years. Data research based on computer-aided diagnosis and texture analysis technology can construct corresponding disease models through tumor heterogeneity, and achieve accurate tumor diagnosis, curative effect and prognostic evaluation (8–10). Many kinds of literature have been published about the application of radiomics in the study of focal hepatic lesions (11–13), but there are few reports on the application of radiomics in the diagnosis of focal hepatic lesions based on unenhanced CT images. In this study, we aimed to develop and validate a computer-aided discriminative diagnosis model to distinguish HCC, hepatic metastasis, hepatic hemangioma, hepatic cyst, hepatic adenoma (HA), and hepatic focal nodular hyperplasia (FNH) using noninvasive radiomic features, based on unenhanced CT images, which facilitate patient-orientated treatment and surgery.

## Materials and Methods

### Patients

The present study was approved by the Institutional Review Board of Zhengzhou University and waived the requirement for informed consent due to its retrospective nature. 522 patients with HCC, hepatic metastases, hepatic hemangioma, hepatic cysts, FNH, HA, underwent CT examination at the First Affiliated Hospital of Zhengzhou University from 2016 to 2018, were included.

The following inclusion criteria were used: (1) HCC, hepatic metastases, FNH, and HA are histopathologically confirmed by surgery or liver percutaneous needle biopsy; (2) enhanced CT scan was performed 30 days before surgery or biopsy; (3) liver occupying lesions number was <= 3. The exclusion criteria were as follows: (1) insufficient quality of CT imaging; (2) lesions less than 1.0 cm (to avoid the impact of small volume effect of the lesion); (3) patients with multisource cancer; (4) patients with hepatitis B, cirrhosis, liver injury or liver surgery found on non-contrast CTs. Seventy patients were excluded, which finally resulted in the enrollment of 452 patients: 77 with HCC, 104 with liver metastases, 126 with hepatic hemangioma, 99 with hepatic cysts, 24 with FNH, 22 with HA. All these lesions were included in the test group.

### Reference Standards

Hepatic hemangioma, hepatic cysts were confirmed by referencing radiologic reports by experienced radiologists and adhering to the following criteria (14): hepatic hemangioma (enhanced nodules around the arterial phase, increased centripetal filling during the venous phase, uniform enhancement during the arterial phase, or continuous enhancement during venous phase); hepatic cyst (close to the CT value of water and no apparent contrast enhancement).

Control group: 452 lesions were diagnosed on unenhanced CT images by an attending physician who worked for 5 years and a chief physician who worked for more than 10 years without knowing the histopathological examination results. The diagnostic accuracy was evaluated with histopathological examination results (HCC, hepatic metastases, FNH, HA) and radiologic reports (hepatic hemangioma, hepatic cyst) as the gold standard.

In addition, six kinds of focal hepatic lesions were divided into benign and malignant groups. The benign group included hepatic hemangioma, hepatic cyst, FNH and HA; the malignant group included HCC and hepatic metastases.

### CT Examination

All examinations were acquired on spectral CT scanners (first generation of Discovery CT750 HD scanner; the second generation of the Discovery CT scanner; both from GE Medical Systems). The following specific scanning parameters were adopted: tube voltage of 120 kV, tube current of 240–300 mA, and layer thickness of 5 mm. Enhanced scanning was performed by injecting a non-ionic contrast agent (iodine content 320 g/L) into the cubital vein at 2.5–4.0 mL/s; the total dose calculated was 1.5 mL/kg. After the contrast agent was injected, the arterial phase was scanned 25–30 s, the venous phase was scanned in 60s, and the delayed phase was scanned after 180 s.

### Tumor Segmentation

**Figure 1:**
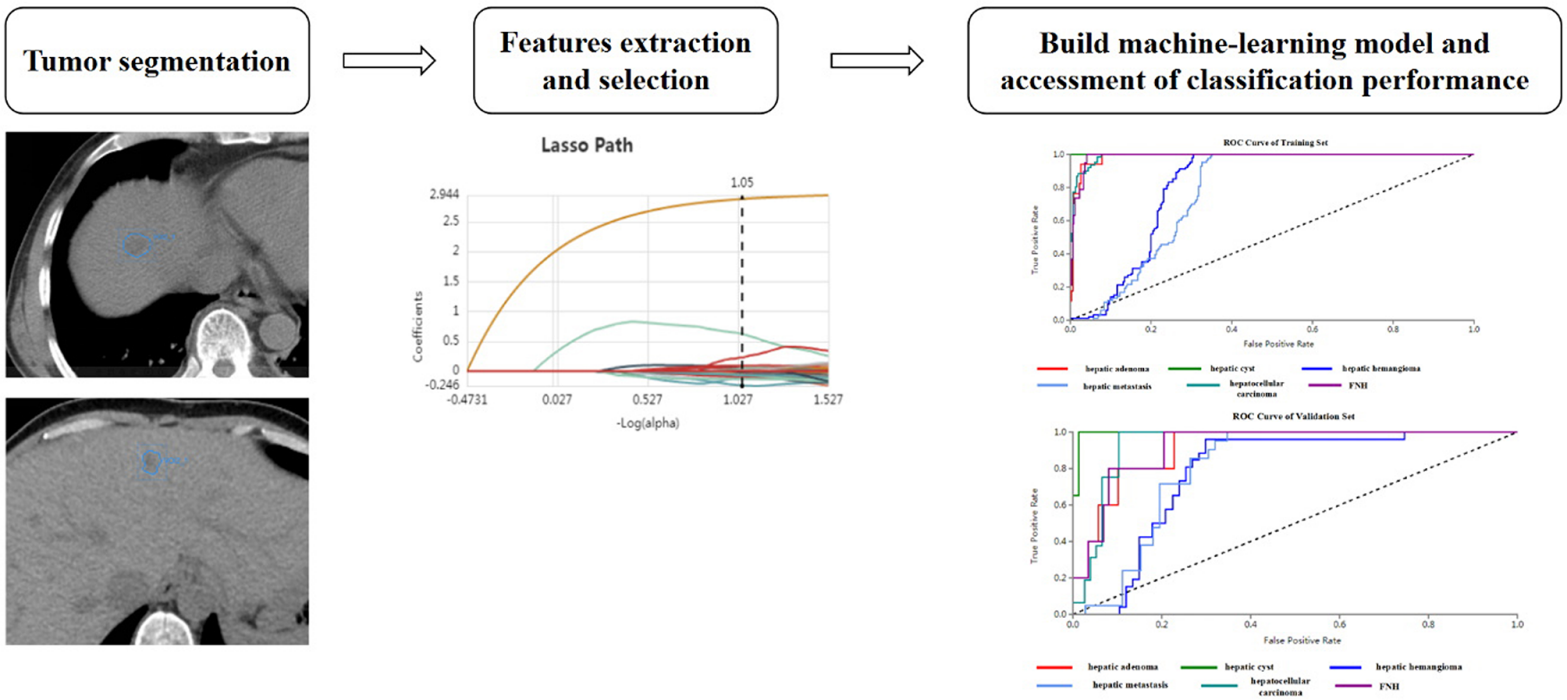
Basic flow chart showing the radiomics method devised for the differential diagnosis of focal hepatic lesions.

Original Digital Imaging and Communications in Medicine (DICOM) images were imported to the Radcloud platform (Huiying Medical Technology Co., Ltd., Beijing, China) for radiomic features extraction and analysis (15). One focal hepatic lesion (not adjacent to the inferior vena cava, caudate lobe, the great hepatic vein, artery, and bile duct) was selected for each patient. Two radiologists manually delineated the region of interest (ROI) along the edge of the lesion, layer by layer, on unenhanced CT images. The volume of interest (VOI) of the lesion was automatically generated by the computer. Another senior radiologist examined the outline results. During the segmentation, corresponding enhanced CT images were referred to determine tumor boundary. Interclass correlation coefficients (ICCs) were used to assessed the interobserver agreement of feature extraction in order to validate the stability of the radiomics features (16). An ICC of greater than 0.75 was considered acceptable. The procedure of radiomics method is illustrated in **Figure 1**.

### Radiomic Features Extraction and Selection

A total number of 1409 quantitative imaging features were extracted from CT images on the Radcloud platform. These features were grouped into four groups. Group 1 (first-order statistics) consisted of 18 descriptors that quantitatively delineated the distribution of the voxel intensities for CT images through commonly used basic metrics. Group 2 (shape- and size-based features) contained 14 three-dimensional features that reflect the shape and size of the region. Calculated from grey-level run-length and grey level co-occurrence texture matrices, 75 textural features that could quantify the region heterogeneity differences were classified into group 3 (texture features). Finally, group 4 (higher-order statistics features) included the first-order statistics, and texture features were derived from wavelet transformation of the original image. We used seven types of filters that included a total number of 14 filters: exponential, gradient, square, square root, logarithm, lbp-2D, and wavelet (wavelet-LLL, wavelet-HHH, wavelet-HLL, wavelet-HHL, wavelet-LLH, wavelet-HLH, wavelet-LHL, and wavelet-LHH).

To explore the generalization performance of the model, training and testing set were split based on a ratio of 8:2 using computer-generated random numbers. All radiomic features were standardized using the Standard Scaler function by removing the mean value, followed by division by its standard deviation and transformation into feature values with zero as the mean and one as the standard deviation.

LASSO is a regression analysis method that can perform both variable selection and regularization to improve the identification accuracy and interpretability of the model. For example, it has a tuning parameter to control the penalty of the linear model, which guarantees the minimum penalty when obtaining a model with a smaller number of features, where the penalty is mean square error (MSE). In addition, another parameter controls the correlation of features, making the selected features less relevant. L1 regularization was used as the cost function, the error value of cross validation was 5, and the maximum number of iterations was 1000. The optimization goal of LASSO is:


$$y=\left(\frac{1}{2*n_{samples}}\right)*{║y-Xw║}^{2}+alpha*║w║y=\left(\frac{1}{2*n_{samples}}\right)*{║y-Xw║}^{2}+alpha*║w║$$

Where *X* is the radioactivity characteristic matrix, *y* is the sample vector marker, n is the sample number, *w* is the coefficient vector regression model, alpha∗║w║ is the lasso punishment.

### Model Building Using Support Vector Machine Classifiers

In this study, we used Support Vector Machine (SVM) classifiers on the Radcloud platform to establish radiomic-based models based on the extracted optimal features. The basic principle of SVM, is to find an optimal hyperplane that produces a better generalization of the dataset. It develops a model that predicts whether a new sample falls into one of the categories or not. Let’s given a training data set *S* = {(*x1, y1*),…,*(xn, yn)*} where *xi* ∈ *Rn* and *y i* ϵ{+1,−1}.

The *xi* represents the transferred input vector and *yi* is the target value. SVM is a binary classifier in which the class labels contain only two values + 1 or −1. From the inputs, SVM draws an optimal hyper-plane *H* that separates the data into different classes and the hyper-plane *H* can be defined as:


xi∈Rn:(w,x)+b=0,w∈Rn,b∈R

The algorithm is based on finding the hyper-plane which gives the maximum distance of separation between training samples using the following function.


f(X)=sign(w,x)+b

For the problem of multiclass learning, SVM solved it as a single multi-class problem further it is modified into multiple binary problems. Hence, the optimal hyper-plane can be combined by the inequality.


$$y_{i}\left\{\left(w,x\right)+b\right\}\geq 1,s.t.i=1,\ldots ,n$$

So, the optimization problem can be written.


$$minimization\frac{1}{2}\left(w^{T},w\right)$$

Notice that the output value of the SVM is either -1 or 1. When the output value of a subimage of suspicious tumor region is 1, the system will classify the tumor in the CT image as a certain category. Conversely, when the output value is -1, the hepatic tumor will be diagnosed as other.

### Statistical Analysis

All statistical analysis were performed with Python 2.7 for Linux. Support vector machine (SVM) were employed based on the optimal discriminative features. Further, the model classification performance was evaluated by AUC (Area Under Curve), accuracy, overall accuracy ((true positives+ true negatives)/(true positives+ false positives+ false negatives+ true negatives)), precision (true positives/(true positives+ false positives)), recall (true positives/(true positives+ false negatives)), f1-score (precision *recall* 2/(precision + recall)), and support (total number in a test set). The formula of confidence interval was as follows:

If 


(n≥30),CI=x±Zα/2×(σ/√n)

If 


(n<30),CI=x±tα/2×(σ/√n)

## Results

### Patients

A retrospective analysis of 452 patients (299 men and 153 women; mean age, 53 ± 14.58 years; range, 3-89 years) was included. The relevant lesions studied included 77 with HCC, 104 with hepatic metastases (40 were lung cancer with hepatic metastasis, 34 were gastric cancer with hepatic metastasis, 30 were colorectal cancer with hepatic metastasis), 126 with hepatic hemangioma, 99 with hepatic cysts, 24 with FNH, 22 with HA.

Control group: The diagnostic accuracy based on unenhanced CT images of the two radiologists was 0.51(231/452) and 0.69 (311/452) respectively.

### Radiomic Features Extraction and Selection

**Figure 2:**
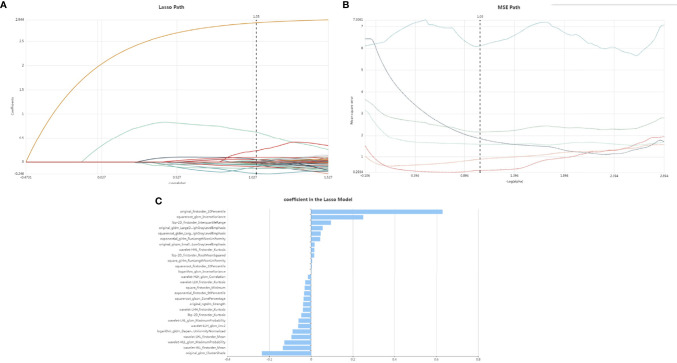
Lasso althorithm on feature selection. **(A)** Lasso path; **(B)** MSE path; **(C)** coefficients in Lasso model. With Lasso model, 27 features were selected according to the optimal alpha.

Our feature stability analysis showed that the ICC for most of radiomics feature was high (**Supplementary Tables 4, 5**). Therefore, all outcomes were based on the measurements of the first radiologist. In the present study, 1409 texture parameters were obtained from each patient. The LASSO algorithm (**Figure 2**) was used to evaluate the correlations between each pair of texture parameters and reduce the dimensionality of the above high-dimensional features based on the optimal λ parameters, and the optimal discriminative features were screened. The final optimal discriminative feature set was comprised of 6 first-order statistics, 1 shape, 8 texture, and 11 higher-order statistics features, a total number of 27 (**Table 1**).

**Table 1:** Description of the selected radiomic features with their associated feature group and filter.

Radiomic feature	Radiomic group	Associated filter
10Percentile	First order	original
Cluster Shade	glcm	original
Large Dependence High Gray Level Emphasis	gldm	original
Small Area Low Gray Level Emphasis	glszm	original
Strength	ngtdm	original
Inverse Variance	glcm	logarithm
Dependence Non Uniformity Normalized	gldm	logarithm
90Percentile	First order	exponential
Run Length Non Uniformity	glrlm	exponential
Minimum	First order	square
Run Length Non Uniformity	glrlm	square
10Percentile	First order	squareroot
Inverse Variance	glcm	squareroot
Large Dependence High Gray Level Emphasis	gldm	squareroot
Zone Percentage	glszm	squareroot
Interquartile Range	First order	lbp-2D
Root Mean Squared	First order	lbp-2D
Kurtosis	First order	lbp-2D
Mean	First order	wavelet-LHL
Maximum Probability	glcm	wavelet-LHL
Kurtosis	First order	wavelet-LHH
Mean	First order	wavelet-HLL
Maximum Probability	glcm	wavelet-HLL
Kurtosis	First order	wavelet-LLH
Imc2	glcm	wavelet-LLH
Correlation	glcm	wavelet-HLH
Kurtosis	First order	wavelet-HHL

GLCM, Gray-level Co-occurrence Matrix; GLDM, Gray Level Dependence Matrix; GLRLM, Gray Level Run Length Matrix; GLSZM, Gray-Level Size Zone Matrix; NGTDM, Neighbouring Gray Tone Difference Matrix.

### Diagnostic Performance of Radiomics Models Among Six Focal Hepatic Lesions

**Figure 3:**
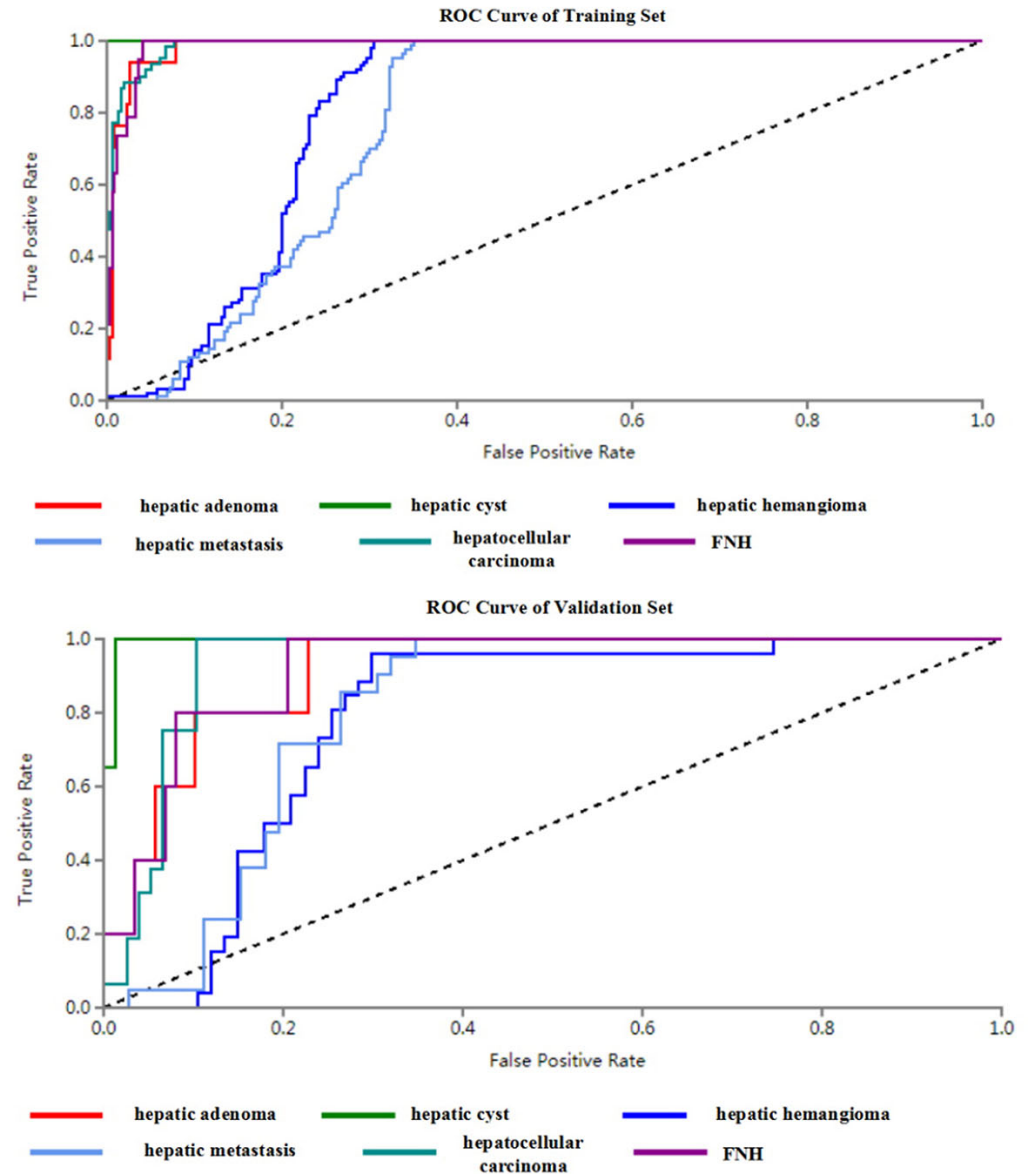
ROC curves of SVM methods to classification (six kinds of focal hepatic lesions).

**Figure 4:**
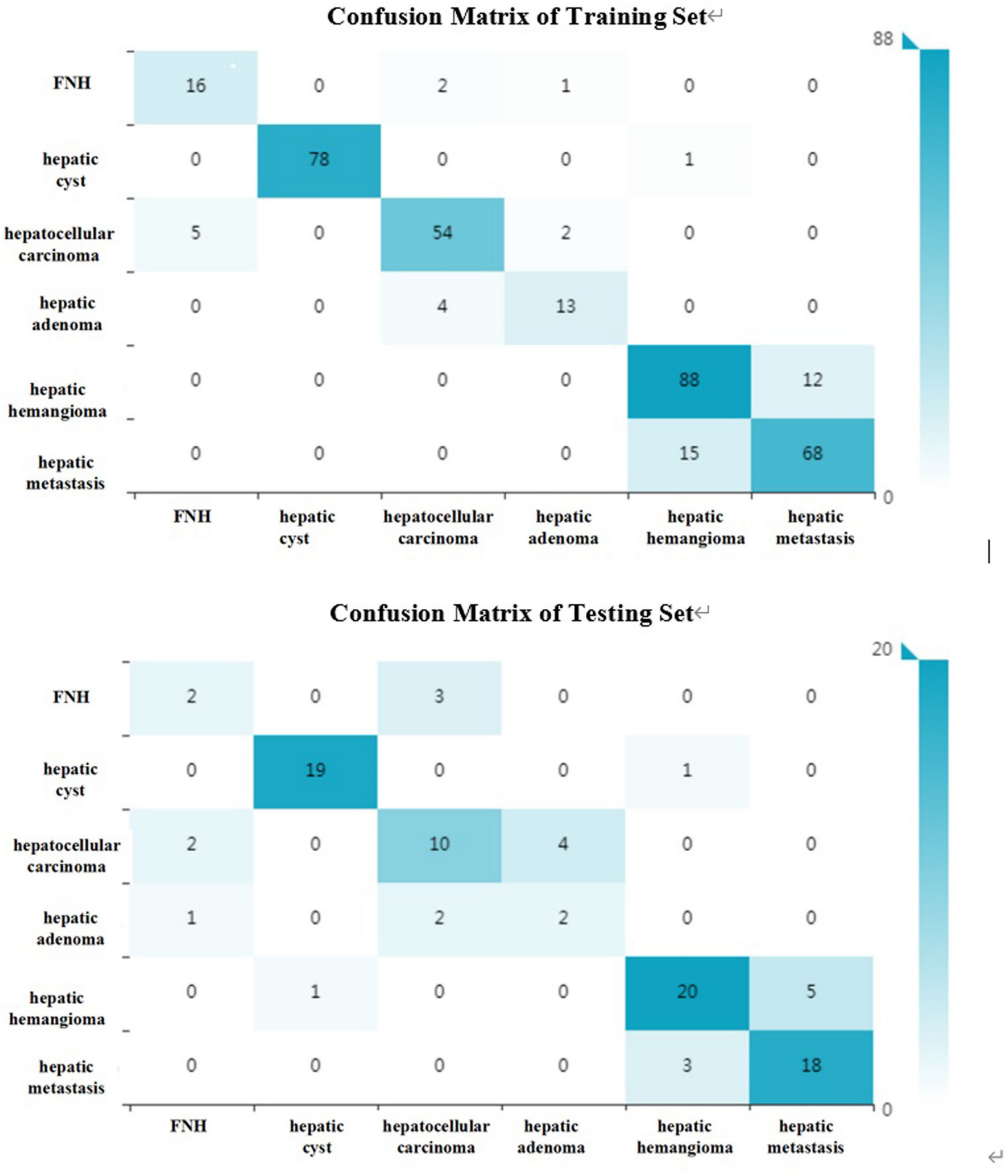
The confusion matrix (six kinds of focal hepatic lesions). Label: overall accuracy (OA) = (true positives+ true negatives)/(true positives+ false positives+ false negatives+ true negatives).

The diagnostic efficacies of SVM for the training set and testing set are presented in **Table 2**. Generally, SVM attained a satisfactory classification performance with an AUC range within 0.942∼1 in the training set and 0.897∼0.995 in the testing set (**Figure 3**). The overall accuracy in the training and testing set were 0.88 and 0.76 (**Table 2**, **Figure 4**), respectively. The other four indicators (precision, recall, f1-score, and support) are summarized in **Supplementary Table 1** for the training set and in **Supplementary Table 2** for the testing set.

**Table 2:** Diagnostic performance of radiomics models among six focal hepatic lesions based on unenhanced CT images.

Category	Training set (n=359)					Testing set (n=93)				
	AUC (95%CI)	Sensitivity	Specificity	Accuracy	OA	AUC (95%CI)	Sensitivity	Specificity	Accuracy	OA
**HCC**	0.982 (0.97-0.99)	0.885	0.980	0.964	0.88	0.933 (0.90-0.96)	0.625	0.935	0.882	0.76
**Hepatic Metastasis**	0.942 (0.92-0.96)	0.819	0.956	0.925		0.904 (0.85-0.95)	0.857	0.931	0.914	
**Hepatic Hemangioma**	0.963 (0.95-0.97)	0.880	0.938	0.922		0.897 (0.83-0.96)	0.769	0.940	0.892	
**Hepatic Cysts**	1 (0.99-1.00)	0.987	1	0.997		0.995 (0.99-1.00)	0.950	0.986	0.978	
**Hepatic Adenoma**	0.987 (0.97-1.00)	0.765	0.991	0.981		0.920 (0.86-0.97)	0.400	0.955	0.925	
**FNH**	0.987 (0.97-1.00)	0.842	0.985	0.978		0.923 (0.86-0.98)	0.400	0.966	0.935	

OA, overall accuracy; AUC, area under curve; CI, confidence interval.

### Diagnostic Performance of Radiomics Models Between Benign and Malignant Focal Hepatic Lesions

**Figure 5:**
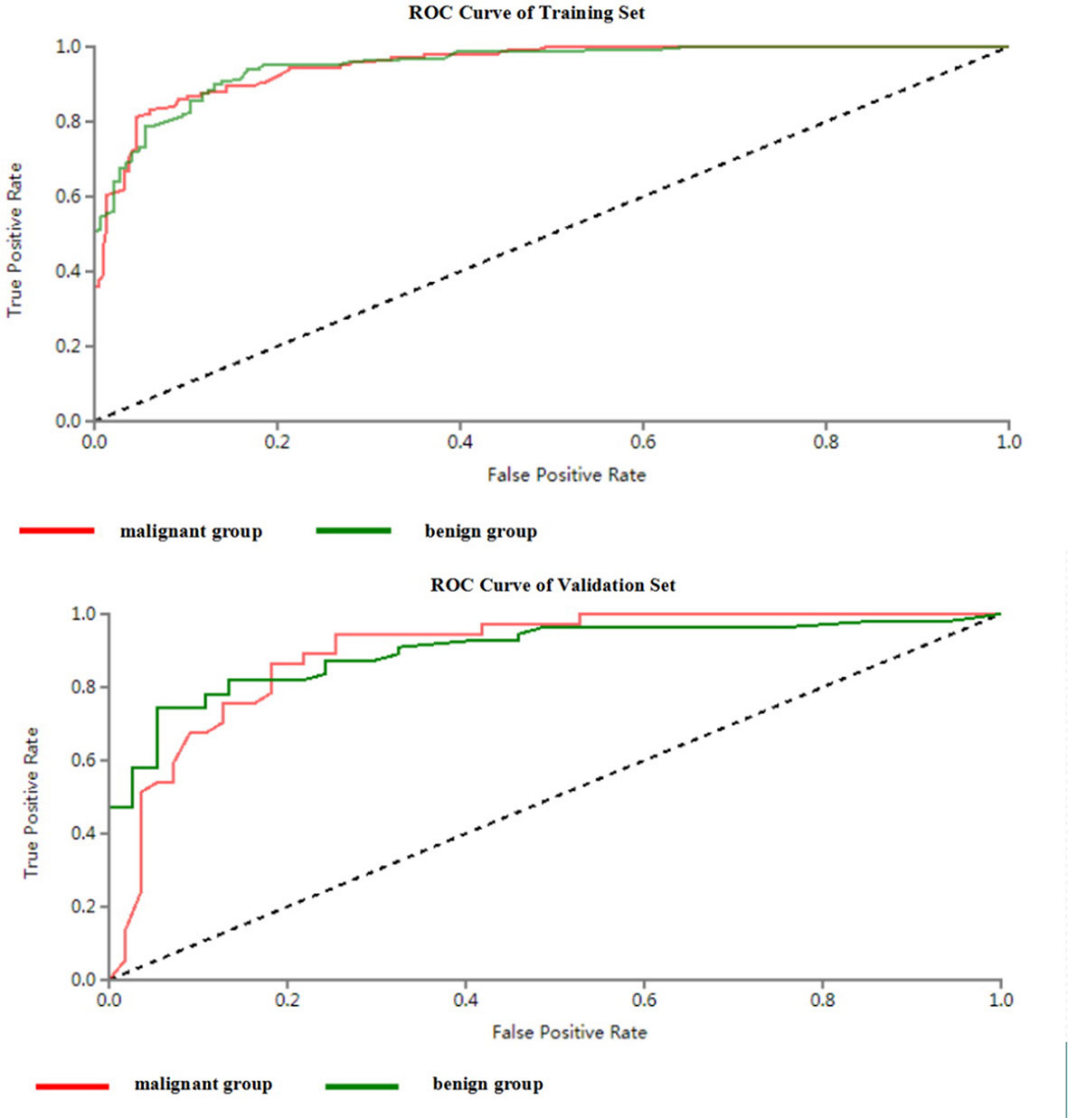
ROC curves of SVM methods to classification (benign and malignant groups).

**Figure 6:**
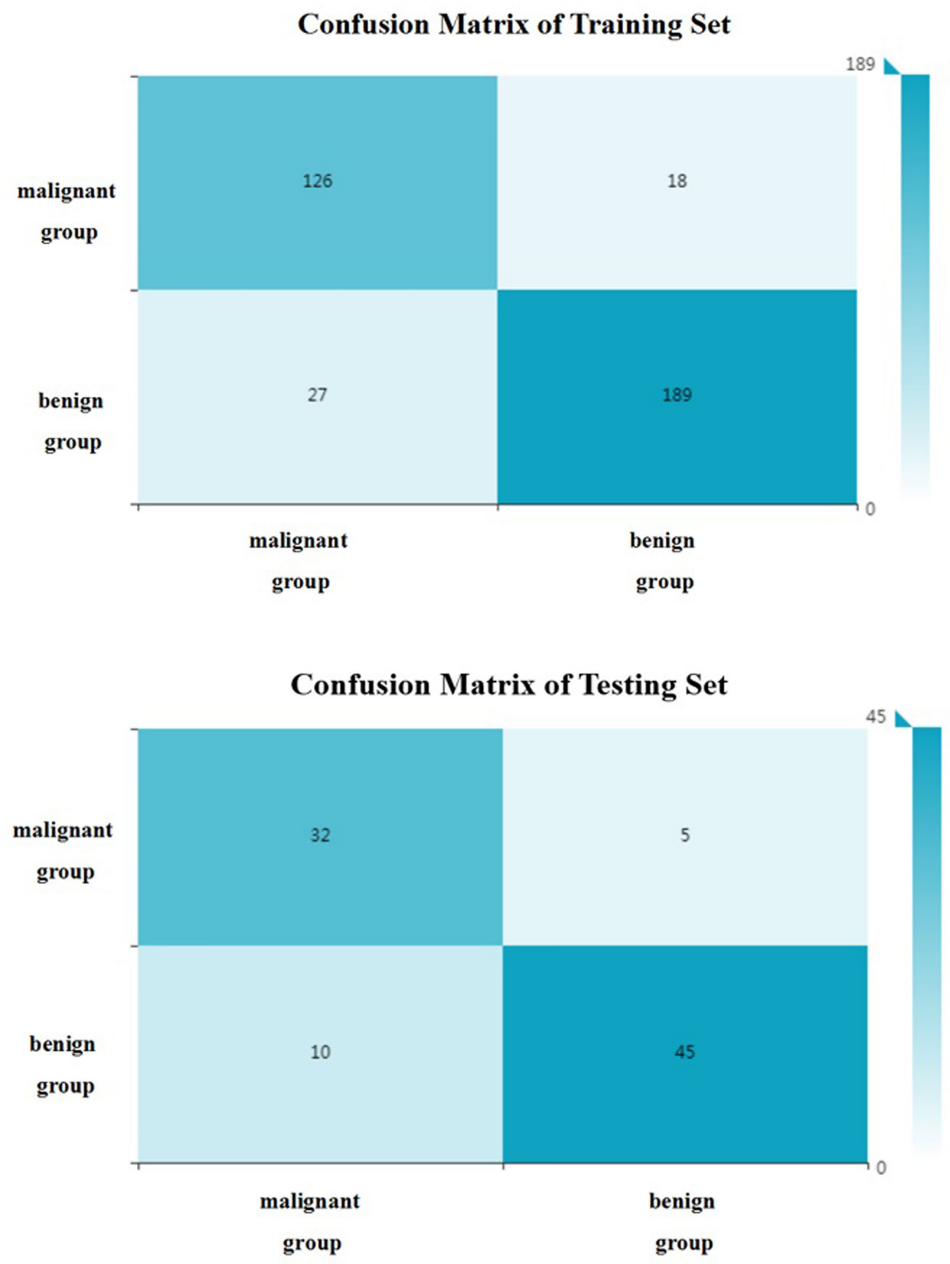
The confusion matrix (benign and malignant groups). Label: overall accuracy (OA) = (true positives+ true negatives)/(true positives+ false positives+ false negatives+ true negatives).

All included patients were divided into benign group and malignant group. The benign group included hepatic hemangioma, hepatic cyst, FNH and HA (272 patients in total); the malignant group included HCC and hepatic metastases (180 patients in total). 34 optimal discriminative features were selected to distinguish the benign and malignant tumors(**Supplementary Table 3**, **Supplementary Figure 1**). Generally, SVM attained a satisfactory classification performance, and the AUC in the training and testing set was 0.951 and 0.899 (**Table 3**, **Figure 5**). The overall accuracy in the training and testing set was 0.89 and 0.84 (**Table 3**, **Figure 6**), respectively.

**Table 3:** Diagnostic performance of radiomics models between benign and malignant focal hepatic lesions.

Category	Training set (n=360)					Testing set (n=92)				
	AUC (95%CI)	Sensitivity	Specificity	Accuracy	OA	AUC (95%CI)	Sensitivity	Specificity	Accuracy	OA
Benign group	0.951 (0.92-0.99)	0.875	0.875	0.875	0.89	0.899 (0.82-0.97)	0.818	0.865	0.837	0.84
Malignant group	0.951 (0.92-0.99)	0.875	0.875	0.875		0.899 (0.82-0.97)	0.865	0.818	0.837	

OA, overall accuracy; AUC, area under curve; CI, confidence interval.

## Discussion

In this study, we developed and validated a novel CAD system based on unenhanced CT images for differentiation among HCC, hepatic metastasis, hepatic hemangioma, hepatic cyst, HA, and FNH, and differentiating benign tumors from malignant tumors. The classifiers all had good diagnostic performance, and the overall accuracies of the training and testing sets were 0.88 and 0.76, 0.89 and 0.84 correspondingly.

As the main examination method for the diagnosis of focal hepatic lesions, enhanced CT has been used in the diagnosis of fatty liver, calcification and focal liver lesions (6, 17–19). However, enhanced CT examination is confronted with the problems of adverse effects caused by contrast agents and radiation doses. Related studies had shown that the incidence of adverse reactions of ionic iodine contrast agents was 12.66%, and the nonionic type was 3.13% (20). And the rate of acute adverse events for low-osmolar contrast agents is approximately 0.2%–0.7% and for severe acute reactions, 0.04% (21). Contrast agent allergy can cause adverse reactions such as nausea and vomiting, dizziness, headache, rash, severe blood pressure, convulsions, shock, and even death (22, 23). Intravenous low-osmolality iodinated contrast material brings some potential risks with a stable estimated glomerular filtration rate less than 30 mL/min/1.73 m^2^, with a trend toward significance at 30–44 mL/min/1.73 m^2^ (24). In addition, Kostas et al. found that the injection of iodine contrast agent could significantly increase the radiation dose of CT irradiation on tissues (25). Therefore, in this study, unenhanced CT images were selected to differentiate focal hepatic lesions, to avoid the disadvantages of contrast agents and to reduce the economic cost.

Radiomics can easily extract high-throughput features from medical images and convert them into quantitative indicators to realize the process transformation from image to data (26). Texture features can not only reflect the morphological features of the lesion visible to the naked eye, but also reflect the microscopic features of the lesion that cannot be observed with the naked eye. It can be more accurate to reflect the heterogeneity of the tumor. Huang et al. used computer-aided diagnosis methods to distinguish liver malignant tumors and liver hemangioma through unenhanced CT images, with an accuracy of 0.817 (27). In the present study, the benign and malignant hepatic diseases were subdivided. Based on unenhanced CT images, the method of texture analysis was used to distinguish HCC, hepatic metastases, hepatic hemangioma, hepatic cyst, hepatic focal nodular hyperplasia, and hepatic adenomas. The overall accuracy of the training set can reach 0.88. And the accuracies of these six kinds of focal hepatic lesions were over 0.85(**Table 2**). The main reason is that this research uses the voxel (VOI) model to extract high-level texture features (1409 in total), which is better than the 2D low-level texture feature model based on the region of interest (ROI). According to the confusion matrix (**Figure 4**), there were 19 focal nodular hyperplasia lesions in the training group; 16 lesions were correctly diagnosed; 2 were misdiagnosed as hepatocellular carcinoma; 1 was misdiagnosed as hepatic adenoma. There was 61 hepatocellular carcinoma; 54 were correctly diagnosed; 5 were misdiagnosed as hepatic focal nodular hyperplasia; 2 were misdiagnosed as hepatic adenomas. There were 17 hepatic adenomas; 13 were correctly diagnosed; 4 were misdiagnosed as liver cancer. Therefore, it is difficult to differentiate atypical focal hepatic nodular hyperplasia, hepatic adenoma and HCC. That might be related to hemorrhagic necrosis (28). The overall accuracy of the testing set was 0.76, lower than the training set, which might be related to the fewer cases of FNH and hepatic adenoma.

The overall accuracy of the training and testing sets in this study is higher than two radiologists. Currently, CT texture analysis studies are undergoing, aimed to facilitate diagnosis by radiologists and help clinicians to make the right therapeutic choice. Although this is only a preliminary study, and a small number of samples of different types of lesions were collected, the results showed that CT texture analysis in combination with appropriate statistical models can be effectively used to identify different lesions.

Differentiation of benign and malignant liver diseases through texture analysis can avoid unnecessary invasive examination and treatment (29). Song et al. selected the arterial phase and used texture analysis to distinguish benign and malignant liver masses (11). the area under the ROC curve can reach 0.927. In the present study, the benign and malignant liver masses were distinguished based on unenhanced CT images. And the area under the ROC curve can reach 0.95 in the training group and 0.90 in the testing set. And according the confusion matrix, the overall accuracy to distinguish the benign and malignant hepatic masses can reach 0.88 and 0.84 separately in the training and testing set. This shows that CT texture analysis has high accuracy in the identification of benign and malignant hepatic masses. Kamel et al. showed that the AUC of dual-source CT in the differential diagnosis of hepatic masses by three experienced radiologists were 0.84, 0.83 and 0.85 respectively (30), which were lower than the results of this study. This shows that texture analysis has better performance in diagnosis. And in the present study, unenhanced CT images were selected to differentiate focal liver lesions, to avoid the disadvantages of contrast agent and reduce the economic cost, so that we can provide another choice for patients who are allergic to contrast agents or can not afford the price of enhanced CT examinations. It makes clinicians have more choices for different patients.

Several limitations of the present study should be acknowledged. Firstly, the sample size is small, especially for focal nodular hyperplasia and hepatic adenoma. In the follow-up study, more new cases should be included and the sample size should be increased; secondly, because of their typical imaging manifestations, patients with hepatic hemangioma and hepatic cyst have not been confirmed by pathology; thirdly, the CT equipment was not uniform. Although 5-mm axial images were selected, the results were compromised due to the different scanning equipment. Fourthly, this is a retrospective single-center study. Further independent prospective multicenter validation cohort and large-scale data are needed to verify the stability of our model; fifthly, patients with hepatitis B, cirrhosis, liver injury or liver surgery found on non-contrast CTs were excluded. Therefore, in these cases, the proposed method can not be applied.

In conclusion, we present a novel tool for potentially distinguishing focal hepatic lesions with noninvasive radiomic features using a computer-aided discriminative diagnosis model based on unenhanced CT images. While our initial results are encouraging, future studies are needed to determine the clinical applications.

## Data Availability Statement

The original contributions presented in the study are included in the article/**Supplementary Material**. Further inquiries can be directed to the corresponding author.

## Ethics Statement

The present study was approved by the Institutional Review Board of Zhengzhou University. Written informed consent to participate in this study was provided by the participants’ legal guardian/next of kin.

## Author Contributions

Study concept and design, XZ, PL, and JG. Imaging data collection, XZ. Imaging preliminary valuation, XZ. Imaging final valuation, PL. Statistical analysis, LY and YJ. Manuscript preparation, XZ. All authors contributed to the article and approved the submitted version.

## Funding

This study has received funding by National Natural and Science Fund of China (NO. 81671682).

## Conflict of Interest

Author YJ was employed by the company Huiying Medical Technology Co., Ltd.

The remaining authors declare that the research was conducted in the absence of any commercial or financial relationships that could be construed as a potential conflict of interest.

## Publisher’s Note

All claims expressed in this article are solely those of the authors and do not necessarily represent those of their affiliated organizations, or those of the publisher, the editors and the reviewers. Any product that may be evaluated in this article, or claim that may be made by its manufacturer, is not guaranteed or endorsed by the publisher.
