# Supplementary material for: Radiomics Study for Differentiating Focal Hepatic Lesions Based on Unenhanced CT Images
Source: Front Oncol. 2022 Apr 27;12:650797. doi: 10.3389/fonc.2022.650797 (PMC9092943; doi:10.3389/fonc.2022.650797)
Supplement: Supplementary file 1 [file DataSheet_1.doc]

**Supplementary Table 1.** The results of four indicators -Precision, Recall, F1-score, Support in training set

|  | **Indicators** | **SVM** |
| --- | --- | --- |
| Hepatic Adenoma | Precision | 0.81 |
|  | Recall | 0.76 |
|  | F1-score | 0.79 |
|  | Support | 17 |
| Hepatic Cysts | Precision | 1.00 |
|  | Recall | 0.99 |
|  | F1-score | 0.99 |
|  | Support | 79 |
| Hepatic Hemangioma | Precision | 0.85 |
|  | Recall | 0.88 |
|  | F1-score | 0.86 |
|  | Support | 100 |
| Hepatocellular Carcinoma | Precision | 0.85 |
|  | Recall | 0.82 |
|  | F1-score | 0.83 |
|  | Support | 83 |
| Hepatic Metastasis | Precision | 0.90 |
|  | Recall | 0.89 |
|  | F1-score | 0.89 |
|  | Support | 61 |
| Focal Nodular Hyperplasia | Precision | 0.76 |
|  | Recall | 0.84 |
|  | F1-score | 0.80 |
|  | Support | 19 |

**Supplementary Table 2.** The results of four indicators -Precision, Recall, F1-score, Support in testing set

|  | **Indicators** | **SVM** |
| --- | --- | --- |
| Hepatic Adenoma | Precision | 0.33 |
|  | Recall | 0.40 |
|  | F1-score | 0.36 |
|  | Support | 5 |
| Hepatic Cysts | Precision | 0.95 |
|  | Recall | 0.95 |
|  | F1-score | 0.95 |
|  | Support | 20 |
| Hepatic Hemangioma | Precision | 0.83 |
|  | Recall | 0.77 |
|  | F1-score | 0.80 |
|  | Support | 26 |
| Hepatic Metastasis | Precision | 0.78 |
|  | Recall | 0.86 |
|  | F1-score | 0.82 |
|  | Support | 21 |
| Hepatocellular Carcinoma | Precision | 0.67 |
|  | Recall | 0.62 |
|  | F1-score | 0.65 |
|  | Support | 16 |
| Focal Nodular Hyperplasia | Precision | 0.40 |
|  | Recall | 0.40 |
|  | F1-score | 0.40 |
|  | Support | 5 |

**Supplementary Table 3. Description of the selected radiomic features with their associated feature group and filter (benign and malignant groups)**

| **Radiomic feature** | **Radiomic class** | **Filter** |
| --- | --- | --- |
| Elongation | shape | original |
| ClusterShade | glcm | original |
| Correlation | glcm | logarithm |
| Skewness | firstorder | exponential |
| Maximum | firstorder | exponential |
| DependenceVariance | gldm | exponential |
| RunLengthNonUniformity | glrlm | exponential |
| GrayLevelNonUniformityNormalized | glrlm | gradient |
| TotalEnergy | firstorder | square |
| Maximum | firstorder | square |
| 90Percentile | firstorder | square |
| DependenceVariance | gldm | square |
| RunLengthNonUniformity | glrlm | square |
| LargeDependenceHighGrayLevelEmphasis | gldm | squareroot |
| GrayLevelVariance | glrlm | squareroot |
| 90Percentile | firstorder | lbp-2D |
| DependenceVariance | gldm | lbp-2D |
| RunLengthNonUniformity | glrlm | lbp-2D |
| Kurtosis | firstorder | wavelet-LHH |
| Mean | firstorder | wavelet-HLL |
| MaximumProbability | glcm | wavelet-HLL |
| Kurtosis | firstorder | wavelet-LLH |
| JointEnergy | glcm | wavelet-LLH |
| GrayLevelNonUniformityNormalized | glrlm | wavelet-LLH |
| LongRunHighGrayLevelEmphasis | glrlm | wavelet-LLH |
| SizeZoneNonUniformityNormalized | glszm | wavelet-LLH |
| Skewness | firstorder | wavelet-HLH |
| Imc2 | glcm | wavelet-HLH |
| Contrast | ngtdm | wavelet-HLH |
| SmallAreaEmphasis | glszm | wavelet-HHH |
| Skewness | firstorder | wavelet-HHL |
| TotalEnergy | firstorder | wavelet-HHL |
| Kurtosis | firstorder | wavelet-HHL |
| Imc2 | glcm | wavelet-HHL |

Label: GLCM=Gray-level Co-occurrence Matrix,GLDM=Gray Level Dependence Matrix,GLRLM=Gray Level Run Length Matrix,GLSZM=Gray-Level Size Zone Matrix,NGTDM=Neighbouring Gray Tone Difference Matrix

**Supplementary Figure 1.** Lasso althorithm on feature selection. **(a)** Lasso path; **(b)** MSE path; **(c)** coefficients in Lasso model. With Lasso model, 34 features were selected according to the optimal alpha.


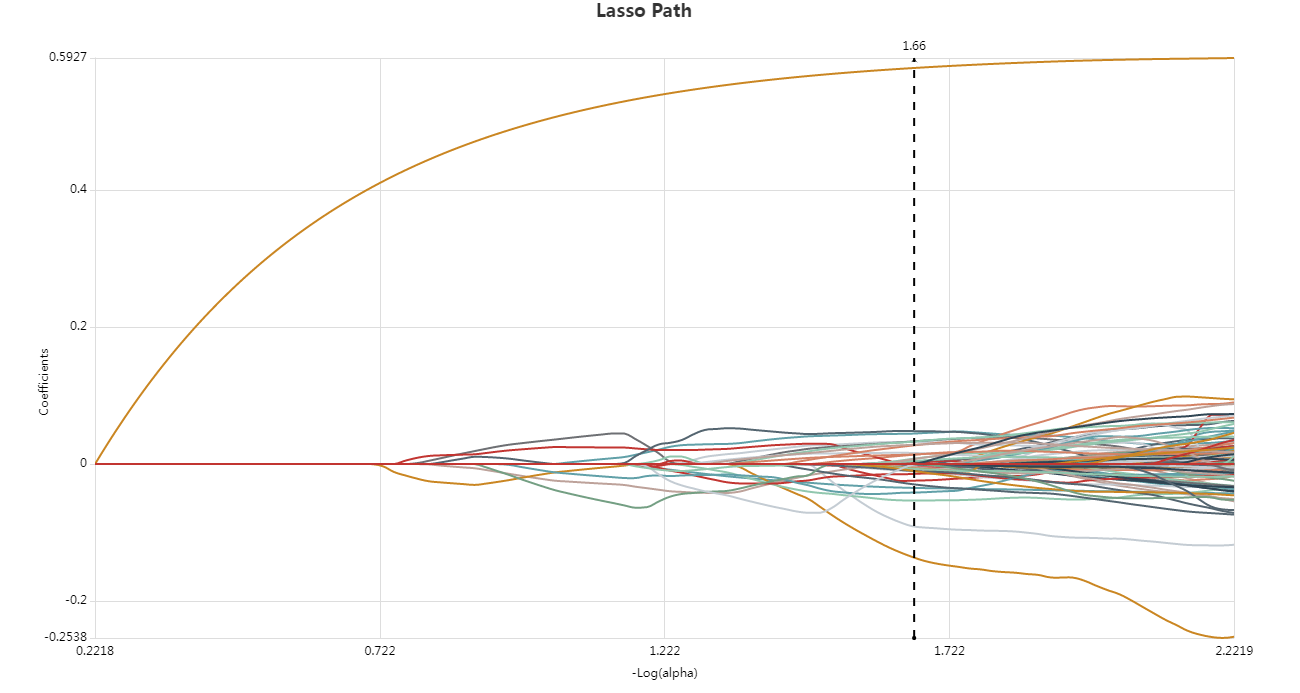
**a**


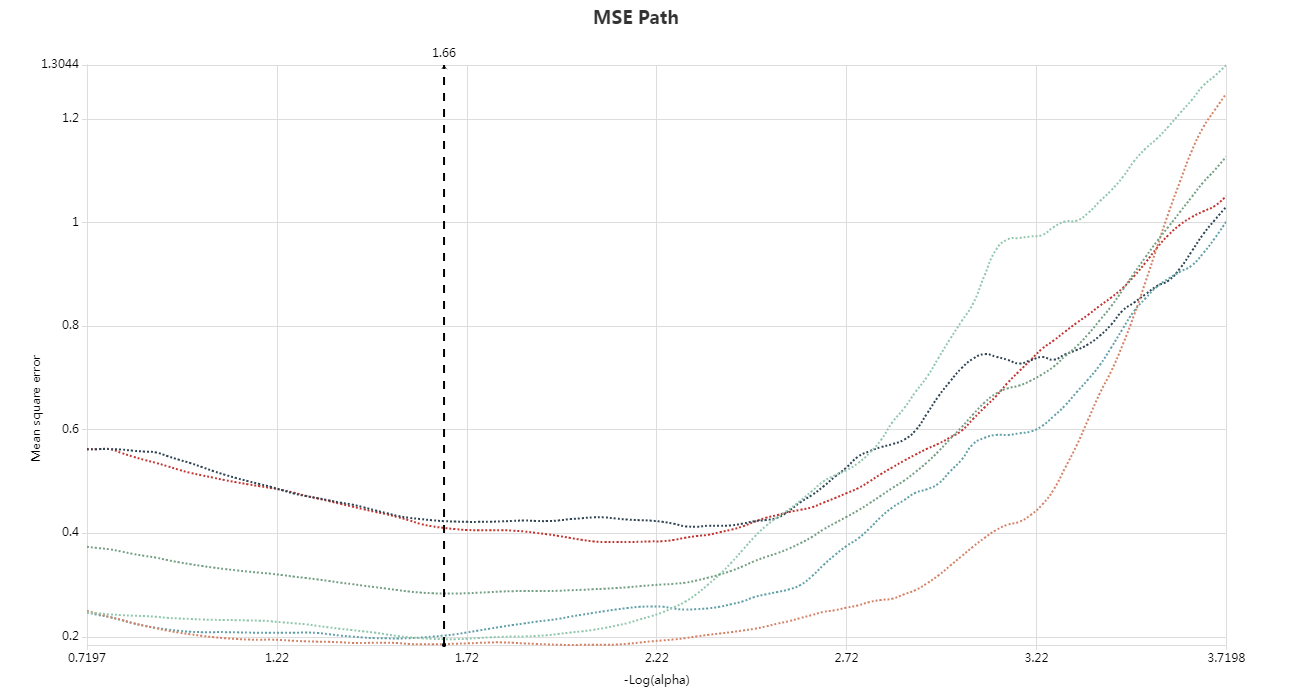
**b**
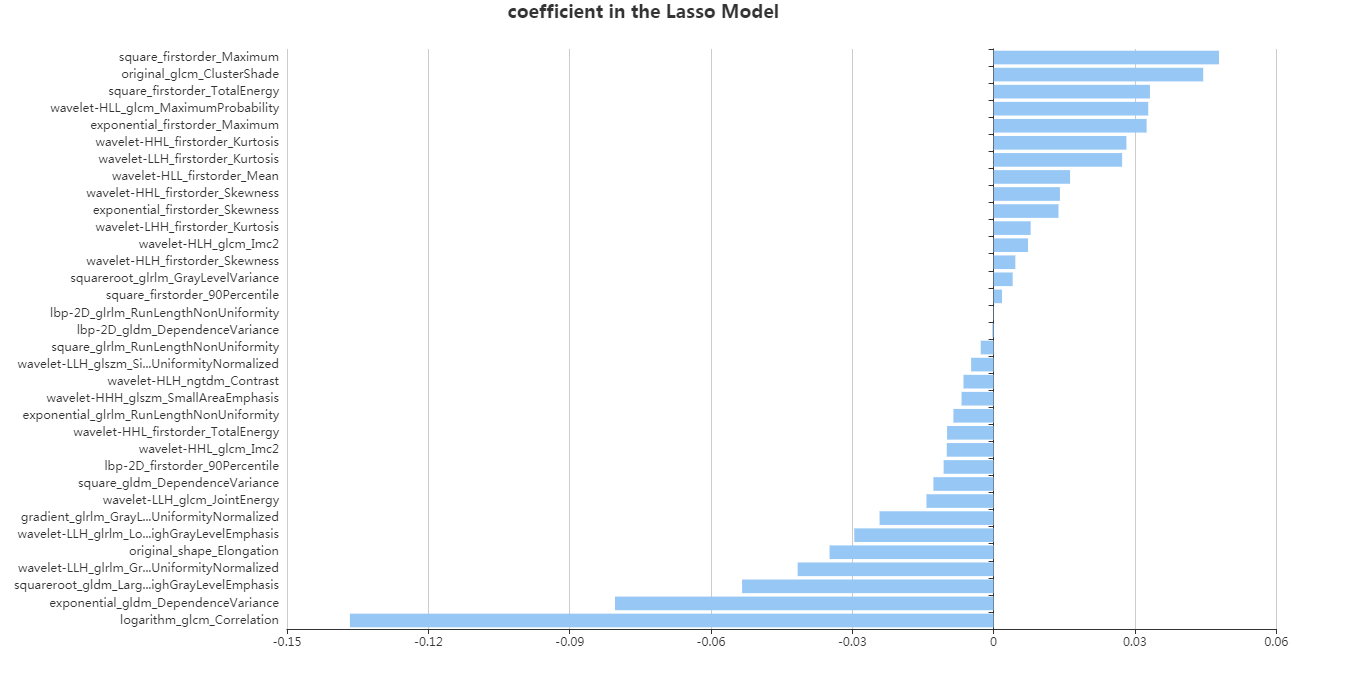
**c**

**Supplementary Table 4.** Inter-observer reproducibility of radiomics features of six focal hepatic lesions

| **Radiomic feature** | **Radiomic group** | **Associated filter** | **ICC** |
| --- | --- | --- | --- |
| 10Percentile | First order | original | 0.998 |
| Cluster Shade | glcm | original | 0.983 |
| Large Dependence High Gray Level Emphasis | gldm | original | 0.998 |
| Small Area Low Gray Level Emphasis | glszm | original | 0.983 |
| Strength | ngtdm | original | 0.980 |
| Inverse Variance | glcm | logarithm | 0.979 |
| Dependence Non Uniformity Normalized | gldm | logarithm | 0.992 |
| 90Percentile | First order | exponential | 0.996 |
| Run Length Non Uniformity | glrlm | exponential | 0.915 |
| Minimum | First order | square | 0.988 |
| Run Length Non Uniformity | glrlm | square | 0.978 |
| 10Percentile | First order | squareroot | 0.996 |
| Inverse Variance | glcm | squareroot | 0.987 |
| Large Dependence High Gray Level Emphasis | gldm | squareroot | 0.998 |
| Zone Percentage | glszm | squareroot | 0.997 |
| Interquartile Range | First order | lbp-2D | 0.979 |
| Root Mean Squared | First order | lbp-2D | 0.992 |
| Kurtosis | First order | lbp-2D | 0.989 |
| Mean | First order | wavelet-LHL | 0.997 |
| Maximum Probability | glcm | wavelet-LHL | 0.977 |
| Kurtosis | First order | wavelet-LHH | 0.883 |
| Mean | First order | wavelet-HLL | 0.979 |
| Maximum Probability | glcm | wavelet-HLL | 0.977 |
| Kurtosis | First order | wavelet-LLH | 0.972 |
| Imc2 | glcm | wavelet-LLH | 0.981 |
| Correlation | glcm | wavelet-HLH | 0.991 |
| Kurtosis | First order | wavelet-HHL | 0.910 |

Label: ICC = Interclass correlation coefficients

**Supplementary Table 5.** Inter-observer reproducibility of radiomics features of benign and malignant focal hepatic lesions

| **Radiomic feature** | **Radiomic class** | **Filter** | **ICC** |
| --- | --- | --- | --- |
| Elongation | shape | original | 0.962 |
| ClusterShade | glcm | original | 0.983 |
| Correlation | glcm | logarithm | 0.974 |
| Skewness | firstorder | exponential | 0.973 |
| Maximum | firstorder | exponential | 0.996 |
| DependenceVariance | gldm | exponential | 0.994 |
| RunLengthNonUniformity | glrlm | exponential | 0.915 |
| GrayLevelNonUniformityNormalized | glrlm | gradient | 0.900 |
| TotalEnergy | firstorder | square | 0.910 |
| Maximum | firstorder | square | 0.993 |
| 90Percentile | firstorder | square | 0.992 |
| DependenceVariance | gldm | square | 0.915 |
| RunLengthNonUniformity | glrlm | square | 0.978 |
| LargeDependenceHighGrayLevelEmphasis | gldm | squareroot | 0.998 |
| GrayLevelVariance | glrlm | squareroot | 0.954 |
| 90Percentile | firstorder | lbp-2D | 0.950 |
| DependenceVariance | gldm | lbp-2D | 0.994 |
| RunLengthNonUniformity | glrlm | lbp-2D | 0.915 |
| Kurtosis | firstorder | wavelet-LHH | 0.883 |
| Mean | firstorder | wavelet-HLL | 0.979 |
| MaximumProbability | glcm | wavelet-HLL | 0.977 |
| Kurtosis | firstorder | wavelet-LLH | 0.972 |
| JointEnergy | glcm | wavelet-LLH | 0.992 |
| GrayLevelNonUniformityNormalized | glrlm | wavelet-LLH | 0.939 |
| LongRunHighGrayLevelEmphasis | glrlm | wavelet-LLH | 0.995 |
| SizeZoneNonUniformityNormalized | glszm | wavelet-LLH | 0.815 |
| Skewness | firstorder | wavelet-HLH | 0.957 |
| Imc2 | glcm | wavelet-HLH | 0.988 |
| Contrast | ngtdm | wavelet-HLH | 0.990 |
| SmallAreaEmphasis | glszm | wavelet-HHH | 0.983 |
| Skewness | firstorder | wavelet-HHL | 0.789 |
| TotalEnergy | firstorder | wavelet-HHL | 0.987 |
| Kurtosis | firstorder | wavelet-HHL | 0.910 |
| Imc2 | glcm | wavelet-HHL | 0.980 |

Label: ICC = Interclass correlation coefficients
